# Safety aspects of using iodoform as a methane-mitigating feed additive to dairy cows: Impacts of milk and serum on bovine mammary and human intestinal epithelial cell lines

**DOI:** 10.3168/jdsc.2025-1048

**Published:** 2026-08-06

**Authors:** Marie Rønn, Mirka Thorsteinsson, Mette Olaf Nielsen, Stig Purup

**Affiliations:** 1 Department of Animal and Veterinary Sciences, Research Centre Foulum, Aarhus University Viborg, DK-8830 Tjele, Denmark; 2 SEGES Innovation, Agro Food Park 15, DK-8200 Aarhus, Denmark

## Abstract

Ongoing research investigates the use of bromoform and other brominated halomethanes as antimethanogenic additives for ruminant livestock. Recently, iodoform has also been shown to be a potent methane inhibitor in dairy cows. To evaluate potential associated effects of using iodoform as a feed additive, cell viability assays were conducted using 2 cell lines: bovine mammary epithelial cells (MAC-T) and human intestinal epithelial cells (FHs 74 Int.), representing mammary and intestinal epithelial cell models, respectively. Cells were cultivated in media containing 1%, 5%, or 10% (vol/vol) of milk whey or blood serum sampled from multicannulated (ruminal, duodenal, and ileal) Danish Holstein dairy cows that were dosed intraruminally daily with iodoform (0, 320, 640, and 800 mg/d). The iodoform dosages were determined based on a previous unpublished pilot study. Moreover, we also investigated the effects of the direct addition of 7 different concentrations (0.003–10 µ*M*) of the pure compounds iodoform, diiodomethane, and iodomethane on the viability of MAC-T cells. Cell viability was increased in both MAC-T and FHs 74 Int. cell lines with increased concentration of serum or whey, whether from control (0 mg/d) or iodoform-supplemented cows. No significant linear or quadratic effects of serum or whey were observed on either cell line. Consistent with these findings, MAC-T cell viability was unaffected by exposure to iodoform or its reduced form, iodomethane, across concentrations of 0.003 to 7 µ*M*. In contrast, diiodomethane reduced cell viability at concentrations >0.5 µ*M*; however, these levels exceed those expected in vivo following iodoform supplementation. Overall, intraruminal administration of iodoform to dairy cows was not associated with adverse effects of serum or whey on MAC-T or FHs 74 Int. cell viability.

## Graphical Abstract

**Figure:**
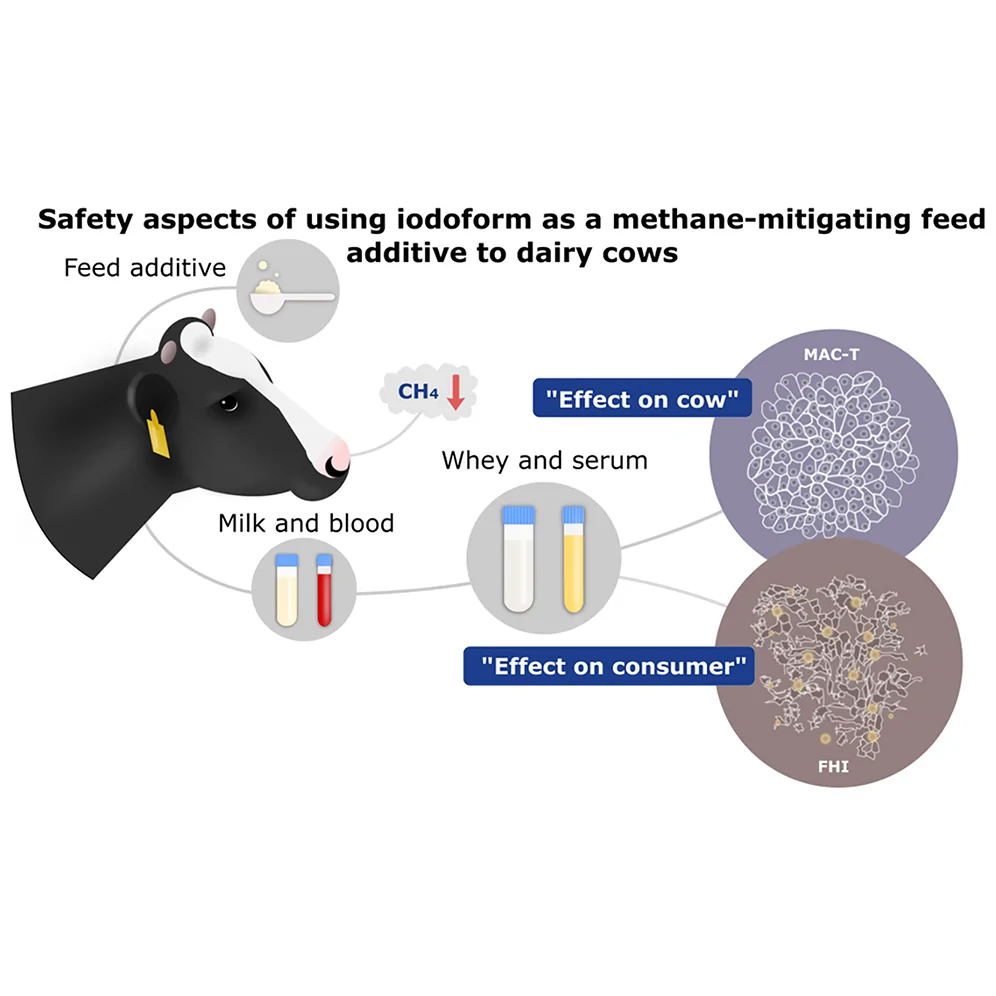

Interest has intensified in trihalomethanes (**THM**), including chloroform, bromoform, bromodichloromethane, and dibromochloromethane, in relation to cattle nutrition, as they are known to exhibit great antimethanogenic properties (Glasson et al., 2022). Chloroform, bromoform, bromodichloromethane, and dibromochloromethane have been thoroughly studied in relation to their toxicology and metabolic pathways in nonruminant animals, including humans, as they are the most common halogenated byproducts formed in chlorinated drinking water (Faustino-Rocha et al., 2016). Recently, iodoform was also found to be a potent inhibitor of enteric methane production in dairy cows (Thorsteinsson et al., 2023). However, the health risks and metabolic pathways of tri- and dihalomethanes in ruminants are, in general, not known.

In some cytotoxic studies, iodo-THM have been found to have a higher toxic potential than their brominated and chlorinated analogs, with the toxicity increasing with number of iodo-groups (Richardson et al., 2008). This increase in cytotoxicity is related to the electronegativity of these halogens, which decreases from chloroform to bromoform to iodoform (Glockler, 1959). However, in terms of carcinogenicity, chloroform and bromoform have shown greater risks than iodoform of inducing tumors in bioassays with mice and rats (National Toxicology Program, 1978). A recent study showed that iodoform is rapidly metabolized by the rumen microbiota to diiodomethane and possibly also iodomethane in smaller amounts. Presence of diiodomethane in blood serum, and hence signs of absorption, could only be detected at the highest exposure levels of iodoform, and at those high exposure levels, diiodomethane was observed at trace levels in milk (Rønn et al., 2026). As a first safety assessment of the use of iodoform as a methane-mitigating feed additive for dairy cows, the aim of the present study was to test whether serum or milk collected from dairy cows pulse-dosed intraruminally twice daily with iodoform, affected cell viability in mammary and intestinal epithelial cell models (bovine mammary epithelial cells, MAC-T; and human small intestinal epithelial cells, FHs 74 Int.), representing tissues exposed to systemic circulation and ingested products, respectively. Furthermore, we tested whether direct addition of the pure compounds iodoform, diiodomethane, and iodomethane to the cell culture medium in concentrations up to 50 times higher than that found in biological samples would affect cell viability of MAC-T cells.

The animal experiments were conducted at Aarhus University, AU Viborg–Research Centre Foulum, Denmark, and complied with the guidelines set out by the Danish Ministry of Environment and Food with respect to animal experimentation and care of animals under study (act no. 2028; Danish Ministry of Environment and Food, 2020). The milk and blood serum samples used in the present study were obtained from a dose-response experiment with Danish Holstein dairy cows in which iodoform was pulse-dosed twice daily during morning (0715 h) and evening (1715 h) milking via the rumen cannula. The cow experiment was conducted as a 4 × 4 randomized Latin square design with 4 multicannulated (ruminal, duodenal, and ileal) Danish Holstein dairy cows (2 second-parity and 2 third-parity; BW: 666 ± 60 kg; milk yield: 33.1 ± 2.10 kg/d; DIM: 210 ± 66 d; DMI: 23.0 ± 1.69) and with 4 two-week periods. In the 4 experimental periods, cows received 0, 320, 640, or 800 mg/d iodoform. The iodoform dosages were determined based on a previous unpublished pilot study. Initially, the highest dose was 1,080 mg/d, but due to large decrease in feed intake in the first period, the dose was reduced to 800 mg/d in the remaining 3 periods. Further details about animal housing, animals, and diet are described in Thorsteinsson et al. (2023). During morning (0630 h) and evening milking (1630 h), half of the daily dose of iodoform was dissolved in 20 mL of 99% ethanol and mixed with 0.5 kg of concentrate mixture and administered into the rumen. The experiment used 2 milk samples (d 9 morning milking and d 11 evening milking) along with blood collected in serum evacuated tubes (Greiner Bio-One GmbH, Kremsmünster, Austria) by puncturing of the tail artery or vein on d 8 (0800 h) and d 10 (1400 h) in each experimental period. Raw milk and blood serum samples were stored at −80°C pending analyses. The milk samples were pooled within cow and period, whereafter the fat and casein were removed using the following procedure: The samples were first centrifuged at 3,500 × *g* for 20 min at 4°C and skim milk aspirated. The skim milk was titrated with 5 *M* acetic acid to precipitation at pH 4.2 to 4.6, after which it was centrifuged at 9,000 × *g* for 30 min at 4°C. The acidic whey was neutralized with 3 *M* NaOH and 1 *M* NaOH to pH 7.4 and centrifuged at 9,000 × *g* for 30 min at 4°C. The 2 blood serum samples per cow per period were handled individually by diluting them to a 10% serum solution in a basal medium (**BM**; described later) and filtered using sterile LLG syringe filters with a pore size of 0.22 µm. Neutralized acidified whey and blood serum solutions were stored at −80°C for later use in the in vitro viability assays.

The MAC-T cell line was an immortalized epithelial cell line derived from the bovine mammary gland and was provided by R. M. Akers on May 21, 1997 (Virginia Tech, Blacksburg, VA). The MAC-T bovine mammary epithelial cell line was selected as a well-characterized and widely used in vitro model of the bovine mammary epithelium. Compared with primary mammary epithelial cells, MAC-T cells provide greater experimental reproducibility owing to their immortalized nature, which enables extensive passaging without senescence and reduces the variability associated with repeated isolation of primary cells. In contrast, primary mammary epithelial cells more closely resemble the in vivo physiological state but are limited by a finite lifespan, heterogeneous cell populations, and donor-to-donor variability, which may complicate the interpretation of subtle treatment effects (Huynh et al., 1991; Jedrzejczak and Szatkowska, 2014; Ontsouka et al., 2016). Therefore, MAC-T cells were considered an appropriate and robust model for the objectives of the present study. The MAC-T cells were cultivated in Dulbecco's Modified Eagle Medium (**DMEM**) containing 1,000 mg/L d-glucose and 110 mg/L sodium pyruvate and supplemented with 15 m*M* HEPES, 4 m*M* Glutamax (Thermo Fisher A/S), and 5% heat-inactivated fetal calf serum (**FCS**; Thermo Fisher A/S). For treatment assays, a BM identical to the culture medium but without FCS was used.

The FHs 74 Int. cells (CCL-241 FHs 74 Int.) were purchased from ATCC (Teddington, United Kingdom). Cells were cultivated with DMEM (with 25 m*M* HEPES) supplemented with 5% FCS, 6 mg/L bovine insulin (Merck-Sigma A/S), 30 µg/L human recombinant epidermal growth factor (**EGF**; Austral Biologicals, San Ramon CA), 8% NCTC-135 medium with L-glutamine (National Collection of Type Cultures, Thermo Fisher A/S, Roskilde, Denmark), 0.4 m*M* sodium pyruvate (Thermo Fisher A/S, Roskilde, Denmark), 0.1 g/L oxaloacetate (Thermo Fisher A/S, Roskilde, Denmark), and antibiotic solution (Thermo Fisher A/S, Roskilde, Denmark) containing 3,156 IU/L penicillin and 3.2 mg/L streptomycin. The BM used for FHs 74 Int. treatment tests contained no FCS and lower concentrations of insulin (10 µg/L) and EGF (0.5 µg/L) than the cultivation medium.

The cells were cultivated in monolayers in 25 cm^3^ (MAC-T) and 12.5 cm^3^ (FHs 74 Int.) flasks and incubated at 37°C with 5% CO_2_ and 95% atmospheric air in a humid environment. Both cell lines were passaged or plated for experiments when 80% to 95% confluency was reached. For viability assays, cells were seeded in 96-well plates at a density of 2,000 cells/well in 0.1 mL of cultivation medium per well for 24 h, whereafter the medium was removed. Each well was washed with PBS (Thermo Fisher A/S, Roskilde, Denmark) and the medium was replaced with 0.1 mL BM with desired concentrations of serum or whey. Serum and whey samples were tested in concentrations of 1%, 5%, and 10% vol/vol following the supplementation of FCS.

The direct effects of iodoform, diiodomethane, and iodomethane on cell viability were tested at 7 different concentrations using cultivation medium instead of BM. Iodoform and diiodomethane were purchased as 99% pure compounds, whereas iodomethane was purchased as a solution with a concentration of 2 mg/mL in 75% methanol water (all provided by Merck-Sigma A/S, Søborg, Denmark). The concentrations were 0.003, 0.01, 0.03, 0.1, 0.3, 1.3, 2.5 µ*M* for iodoform; 0.01, 0.05, 0.1, 0.5, 1, 5, 10 µ*M* for diiodomethane; and 0.007, 0.04, 0.4, 0.7, 3.5, 7.1 µ*M* iodomethane. These concentrations correspond to those previously reported in serum and milk (Rønn et al., 2026), as well as levels up to 200-fold higher, to assess potential compound-related toxicity.

All samples, concentrations, and compounds were tested in triplicate wells in triplicate plates, and each plate included negative and positive controls (i.e., cells were cultivated in either BM or in cultivation medium including 5% FCS, respectively). On d 3, cell viability was determined by a resazurin metabolism assay (alamarBlue cell viability reagent, BioSource, AH diagnostic, Aarhus, Denmark). A solution of 100 µL/mL alamarBlue in PBS was added in the amount of 110 µL in each well, and the fluorescence of resorufin was measured on a EnVision XCite Plate Reader (PerkinElmer Inc., Skovlunde, Denmark) at 544 nm (excitation) and 596 nm (emission) after an incubation time of 2 h. The fluorescence measured is proportional to the number of living cells and thereby indirectly a measurement of cell viability.

All data were analyzed in R 4.3.1 (R Core Team, 2023). Analysis of the impacts of whey and serum on viability of MAC-T and FHs 74 Int. cell lines were conducted separately. Viability of cells was analyzed with the following linear mixed effects model using the “lmer” function from the lme4 package (Bates et al., 2015):

*Y_ijklmno_* = *μ* + *α_i_* + *β_j_* + *γ_ij_* + *δ_k_* + *θ_l_* + *τ_m_* + *P_o_*_(_*_km_*_)_ + *C_n_*_(_*_okm_*_)_ + *R_j_*_(_*_lnokm_*_)_ + *ε_ijklmno_*.

The fixed effects were parameterized relative to the reference levels (*i* = *j* = *k* = *l* = *m* = 1). Fixed effects included iodoform dose administered to cows (0, 320, 640, or 800 mg/d), concentration of biological sample added to the cell culture medium (1%, 5%, or 10%, vol/vol), the difference in concentration of biological sample added to media in the cell viability test (1%, 5%, 10% vol/vol), the interaction effect between treatment and concentration, the differences by feeding trial experimental period (1–4), the difference between days of sample collection in each period (d 8 or 10), and the difference from when repeating each cell viability assay (A–C). The letters *P*, *C*, and *R* represent random effects: A is the random effect of assay in the cell viability analysis = 1, 2 with 2 cows per assay; B is accounting for correlations between repeated observations from cow = 1, 2 distributing the 4 cows with 2 on each assay within experimental period in feeding trial within repeated assay from cell culture study; and C are repeated concentrations of biological sample added to media within day, cow, assay, period, and repeated assay = 1, 2, 3. Finally, the residual and the random effects are assumed to be mutually independent and normally distributed. The statistical analyses from cell culture trials with whey were conducted in a similar way, although the sampling day effect was excluded. Treatment was treated as a continuous variable and assessed sequentially for linear and quadratic effects and for interaction between treatment and dose. Nested models were compared using Kenward–Roger adjusted *F*-tests (pbkrtest package) to account for the small sample size.

The effect of treating MAC-T cells to pure compounds of iodoform, diiodomethane, and iodomethane were analyzed similarly with a mixed model in R. In this analysis, *Y_im_* is the cell viability to relative the negative control. The model is as follows:

*Y_im_* = *μ* + *α_i_* + *R_j_*_(_*_m_*_)_ + *ε_im_*.

The fixed effects are parameterized by *μ* for the reference combination (*i* and *m* = 1), *α_i_* is the tested halomethane concentration in medium together with a positive control. *R* represents the random effect of repeated assay, *j*(*m*) = A, B, C. Finally, the residual *ε* and the random effect is assumed to be mutually independent normally distributed with zero mean and variance parameters *σ*^2^ and $\sigma_{R}^{2}.$ All levels of each compound were compared with the positive control (medium with 5% FCS). Significant effects were obtained with *P* ≤ 0.05.

Positive controls (cultivation medium containing 5% FCS) were included on each assay to make sure that cells performed normally. The total fluorescence cell count after 2 d of cultivation for the MAC-T positive controls were on average ∼3,200,000 pixel density units (**p.d.u.**), whereas the average fluorescence cell count for the positive controls with FHs 74 Int. was ∼3,900,000 p.d.u. Under these ideal conditions, both cell lines performed as expected. The effects of increasing concentration of serum and whey on MAC-T and FHs 74 Int. obtained from cows on 0 mg/d treatment are presented in Figure 1, Figure 2. It is evident that both 0 mg/d serum and whey stimulated cell viability in both cell lines compared with cultivation in BM. Upon exposure to whey in the medium, FHs 74 Int. cells increased in relative cell viability from 1% to 10% whey, going from 0.94× BM to 1.65× BM, respectively, whereas MAC-T cells increased from 1.59× BM to 1.94× BM. The FHs 74 Int. cells exposed to serum increased in relative cell viability from 1.17× BM to 4.29× BM when serum concentrations in the medium increased from 1% to 10%, respectively, and the relative viability in MAC-T cells increased from 1.71× BM to 3.70× BM at 1% to 10% serum exposure. This increase is consistent with the presence of growth-promoting components in serum and whey and indicates that 0 mg/d samples supported normal cellular proliferation compared with conditions without proliferation supplements (no FCS).

**Figure 1:**
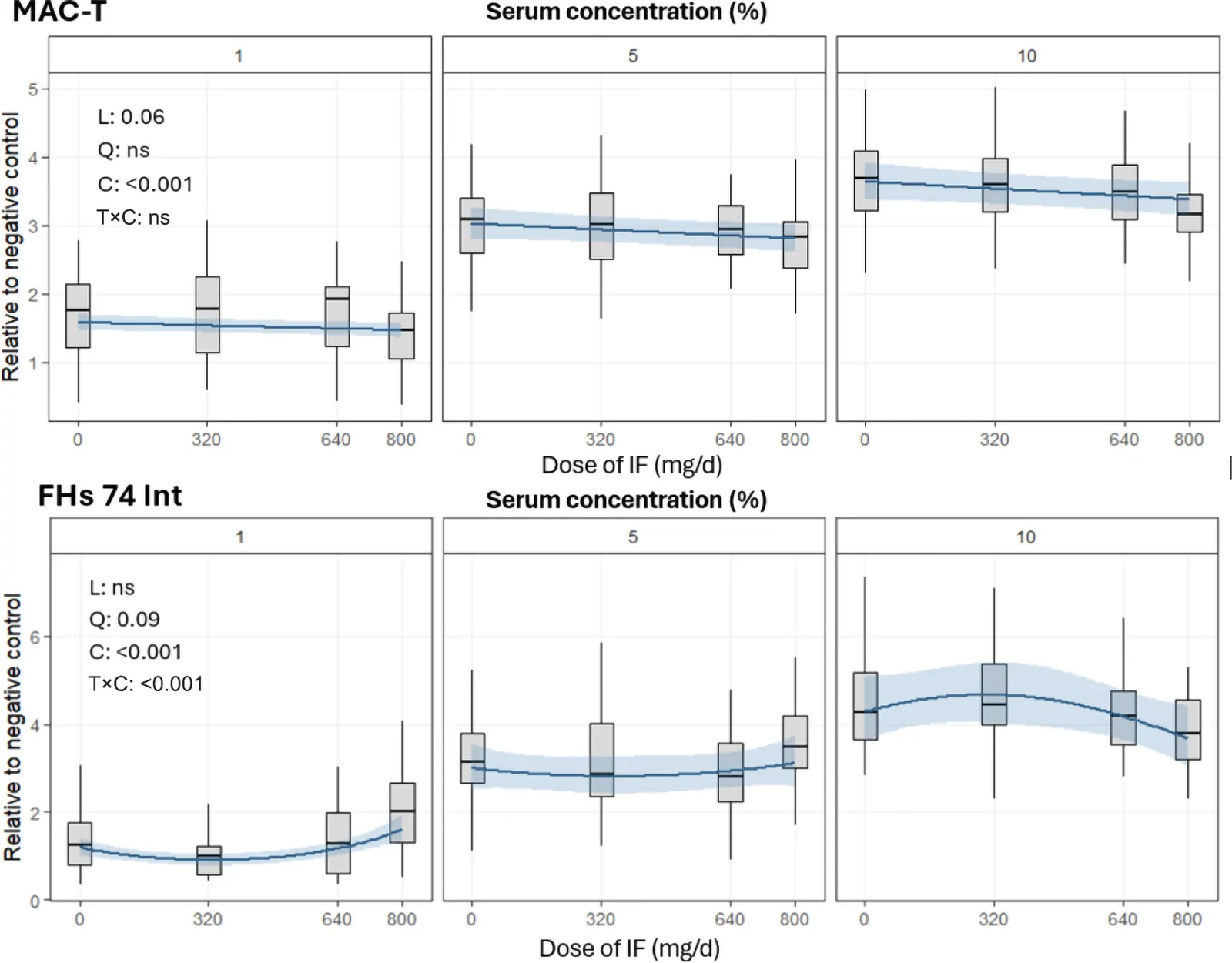
Cell viability, as least mixed model means, expressed relative to values for the negative control (basal medium without fetal calf serum) for MAC-T cells and FHs 74 Int. cell lines exposed to increasing amounts of serum originating from dairy cows receiving 4 different doses of iodoform (IF; 0, 320, 640, and 800 mg/d). The box plots show the median (horizontal line) and the 25th and 75th percentiles (lower and upper edges of the box, respectively), and the whiskers represent minimum and maximum values; the blue area represents the 95% confidence interval. The model was tested for linear (L), quadratic (Q), effect of concentration (C), and interactive effects between treatment and concentration (T×C). ns = not significant.

**Figure 2:**
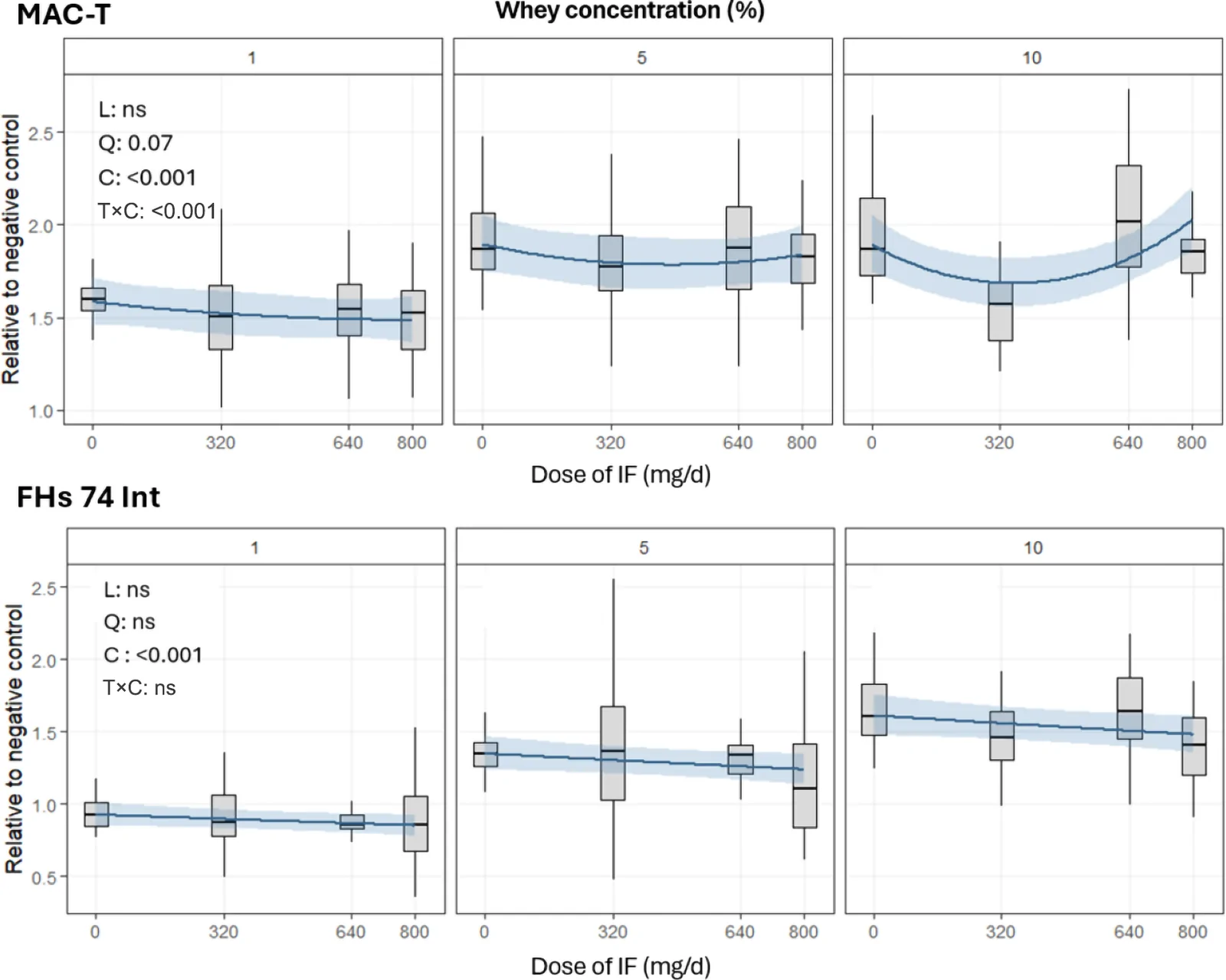
Cell viability, as least mixed model means, expressed as a relative value to negative control (basal medium without fetal calf serum) for MAC-T cells and FHs 74 Int. cell lines exposed to increasing amounts of milk whey originating from dairy cows receiving 4 different doses of iodoform (IF; 0, 320, 640, and 800 mg/d). The box plots show the median (horizontal line) and the 25th and 75th percentiles (lower and upper edges of the box, respectively), and the whiskers represent minimum and maximum values; the blue area represents the 95% confidence interval. The model was tested for linear (L), quadratic (Q), effect of concentration (C), and interactive effects between treatment and concentration (T×C). ns = not significant.

Only tendencies were observed for linear and quadratic effects of dose on the cell viability of MAC-T cells or FHs 74 Int. cells when they were cultivated in media containing serum from iodoform-treated cows (Figure 1, Figure 2). However, 1 cow was removed from the serum dataset of FHs 74 Int. cells, as 0, 320, 640, and 800 mg/d serum samples from this cow inhibited rather than stimulated cell viability. This cell viability response to serum inclusion was not visible with the MAC-T cells exposed to the exact same samples or with serum samples from the other cows on the FHs 74 Int. cell line; it is therefore unknown what caused this effect. The cow was removed to ensure that the massive increase in variation in cell viability caused by this irregularity would not mask any effects in the blood samples from the 3 other cows. However, it must be noted that when 1 cow was excluded from the FHs 74 Int serum dataset, the 800 mg/d dose only included data from 2 cows, as data are missing from 1 cow receiving the highest dose due to dose corrections in the first period.

Because the present tendencies most likely did not originate from the halomethane itself, the biological responses can possibly be explained by other compounds present in the samples. Thorsteinsson et al. (2023) reported increased concentrations of serum nonesterified fatty acid with increasing iodoform dose, indicating that cows entered an increasing stage of negative energy balance on 640 mg/d and 800 mg/d due to decreased DMI. A negative energy balance is normally associated with reduced hepatic synthesis and release of the hormone IGF-1 (Sharma et al., 1994), which is a potent mitogen in mammary epithelial cells (Purup et al., 2000). Furthermore, plasma concentrations of the thyroid hormone thyroxine, T4, decreased linearly with increasing supplementation of iodoform to the cows (Thorsteinsson et al., 2023). Thyroid hormones have been shown to stimulate cell proliferation in rat pituitary tumor germinal center cells (Barrera-Hernandez et al., 1999) and increase lactation intensity by enhancing the responsiveness of other hormones or factors in the mammary cells (Neville et al., 2002). Increased serum levels of bilirubin were also observed with increasing iodoform dose in Thorsteinsson et al. (2023). Although bilirubin is normally considered an indicator of liver health status, awareness has increased over the past decades of its role as an essential constituent of cellular signaling pathways, where bilirubin causes growth inhibition in breast cancer cell lines (MCF-7 and MDA-MB-468) by elevated cell apoptosis (Shahrokhi et al., 2021). Additionally, bilirubin inhibits cell vascular smooth muscle cell proliferation (Öllinger et al., 2005) but has a stimulative effect in a rat kidney cell line (NRK52E) until a threshold point, whereafter it suppresses cell viability and communication (Wang et al., 2013). It is important to note that the concentrations of T4 and bilirubin were within the normal range of healthy cows in Thorsteinsson et al. (2023). However, these changes in concentrations of endocrine and signaling factors in serum are still the most likely explanations for the suppressed stimulation of cell viability at the high concentration of serum in the media from 800 mg/d iodoform–supplemented cows rather than a direct action of iodoform, diiodomethane, or iodomethane.

We found no effects of exposing MAC-T or FHs 74 Int. to milk whey from 320, 640, or 800 mg/d iodoform–treated cows compared with 0 mg/d cows. The whey fraction was chosen because it represents the soluble aqueous phase of milk that is readily accessible to cultured cells. In contrast, fat globules and casein micelles are not well suited for direct evaluation in this in vitro system. Further, the whey fraction contains metabolites, growth factors or hormones, stress-related peptides, and inflammatory markers that are most likely to mediate cellular responses. However, although no dose effects of milk whey or iodoform supplementation were detected, it is important to stress that the preprocessing of milk samples involved skimming. Because THM are lipophilic, iodoform and derived metabolites could therefore have been removed to an unknown extent with the milk fat and hence not retained in the whey added to the culture medium.

In this study, the viability of the MAC-T cell line was assessed following exposure to increasing concentrations of iodoform, diiodomethane, and iodomethane, which were all within the range expected to occur when cows are supplemented with iodoform (Figure 3). As previously reported, there were no detectable levels of iodoform in any biological fluid sampled from the cows used in this study, but diiodomethane was quantifiable in rumen fluid, duodenal fluid, and blood serum (concentrations in serum: 0 and 320 mg/d = 0 µ*M*, 640 mg/d = 0.015 µ*M*, 800 mg/d = 0.019 µ*M*), and trace amounts were also detected in milk and urine (Rønn et al., 2026). Therefore, to support the previously reported results, we implemented the test of adding halomethanes directly to the culture media. This showed that viability of MAC-T cells was not suppressed until the diiodomethane concentration was higher than 0.5 µ*M* (Figure 3). Similarly, the suppressing effects of iodomethane are likely just variations in cell viability, as the suppression did not decrease with increasing concentration. It is therefore unlikely that iodoform itself or the derived diiodomethane (or potentially iodomethane) will have any suppressing effects on cell viability in the expected concentrations present in the exposed cows.

**Figure 3:**
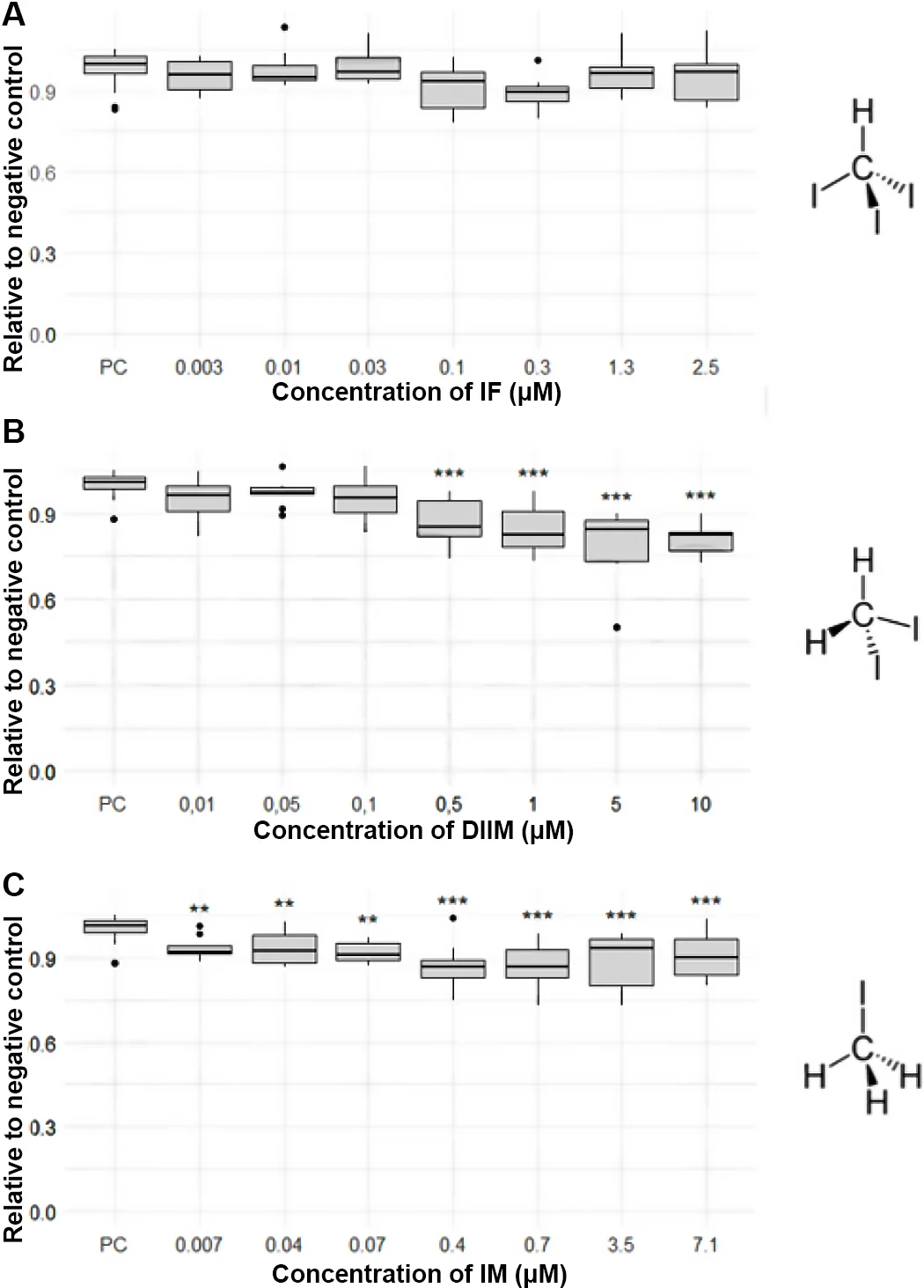
MAC-T exposed to iodoform (IF; A), diiodomethane (DIIM; B), and iodomethane (IM; C). The 3 different halomethanes were mixed into 3 separate stock solutions of cultivation medium (5% fetal calf serum) whereafter pre-cultivated cells were exposed to the halomethanes in the stated concentrations on the x-axis. The cell viability was calculated as a relative value to negative control (basal medium without fetal calf serum). The relative value for each halomethane concentration was compared with positive controls (cultivation medium with 5% FCS) for any reduction in cell viability. The box plots show the median (horizontal line) and the 25th and 75th percentiles (lower and upper edges of the box, respectively), and the whiskers represent minimum and maximum values. *P ≤ 0.05; **P ≤ 0.01; ***P ≤ 0.001.

Even though iodoform did not reduce cell viability, other studies have evaluated its cytotoxic and genotoxic effects in different cell models. In these studies, iodoform was tested on Chinese hamster ovary cells, a commonly used epithelial cell line, and NIH3T3 cells, a murine fibroblast cell line; cytotoxic effects were observed in both cell types, and some unsystematic DNA damage was also reported in NIH3T3 cells (Richardson et al., 2008; Wei et al., 2013). However, cytotoxic effects in Chinese hamster ovary cells were only observed at concentrations exceeding 55 µ*M* (Richardson et al., 2008). Assuming the rumen capacity of a dairy cow is ∼80 L of rumen liquid (Krämer et al., 2013), with instantaneous mixing and no metabolism or absorption of iodoform following direct ruminal administration, the resulting concentration would not have exceeded ∼13 µ*M* at the 800 mg/d treatment level. It is therefore highly unlikely that iodoform concentrations in biological fluids would reach or exceed levels associated with cytotoxic effects in previous studies.

One of the main considerations in cell-based studies is the choice of cell-based models (target tissue or organ). Bovine mammary cells and human intestinal cells were chosen as proxies for a target organ in the cow and consumer when exposed to changes in bioactive components circulating in the blood or being excreted in milk, respectively. MAC-T cells retain several biochemical and morphological characteristics typical of mammary epithelial cells in vivo and therefore represent a general model of the mammary gland. Small intestinal epithelial cells are first targets for bioactive components delivered through milk, as they lie at the interface between dietary components in the intestinal lumen and the internal space of an individual. For studies of milk-derived bioactive compounds, FHs 74 Int. fetal cells have been widely used as a model, as these cells represent a very sensitive model for small intestinal epithelial cells (Purup and Nielsen, 2012). Therefore, these cell lines were chosen for a first assessment of potential impacts for the dairy cow itself and the human consumer of milk when dairy cows are supplemented with iodoform. It is possible that the use of other cell lines in the present study would have demonstrated other effects.

In conclusion, serum and whey from dairy cows receiving daily intraruminal iodoform supplementation did not affect the viability of MAC-T or FHs 74 Int. cells. This is supported by the absence of effects of both iodoform and iodomethane on the cell viability of MAC-T. Although diiodomethane reduced viability at high concentrations, these levels were substantially above those expected in vivo following iodoform supplementation, indicating that such effects are not biologically relevant under practical conditions.
